# Using the Knowledge to Action Framework in practice: a citation analysis and systematic review

**DOI:** 10.1186/s13012-014-0172-2

**Published:** 2014-11-23

**Authors:** Becky Field, Andrew Booth, Irene Ilott, Kate Gerrish

**Affiliations:** School of Health and Related Research (ScHARR), University of Sheffield, Sheffield, UK; NIHR CLAHRC SY, Sheffield, UK; School of Nursing and Midwifery, University of Sheffield, Sheffield, UK; Sheffield Teaching Hospitals NHS Foundation Trust, Sheffield, UK; NIHR CLAHRC YH, Sheffield, UK

**Keywords:** Knowledge translation, Conceptual framework, Systematic review, Implementation, Citation analysis, Knowledge to Action Framework, Theory, Theory fidelity

## Abstract

**Background:**

Conceptual frameworks are recommended as a way of applying theory to enhance implementation efforts. The Knowledge to Action (KTA) Framework was developed in Canada by Graham and colleagues in the 2000s, following a review of 31 planned action theories. The framework has two components: Knowledge Creation and an Action Cycle, each of which comprises multiple phases. This review sought to answer two questions: ‘Is the KTA Framework used in practice? And if so, how?’

**Methods:**

This study is a citation analysis and systematic review. The index citation for the original paper was identified on three databases—Web of Science, Scopus and Google Scholar—with the facility for citation searching. Limitations of English language and year of publication 2006-June 2013 were set. A taxonomy categorising the continuum of usage was developed. Only studies applying the framework to implementation projects were included. Data were extracted and mapped against each phase of the framework for studies where it was integral to the implementation project.

**Results:**

The citation search yielded 1,787 records. A total of 1,057 titles and abstracts were screened. One hundred and forty-six studies described usage to varying degrees, ranging from referenced to integrated. In ten studies, the KTA Framework was integral to the design, delivery and evaluation of the implementation activities. All ten described using the Action Cycle and seven referred to Knowledge Creation. The KTA Framework was enacted in different health care and academic settings with projects targeted at patients, the public, and nursing and allied health professionals.

**Conclusions:**

The KTA Framework is being used in practice with varying degrees of completeness. It is frequently cited, with usage ranging from simple attribution via a reference, through informing planning, to making an intellectual contribution. When the framework was integral to knowledge translation, it guided action in idiosyncratic ways and there was theory fidelity. Prevailing wisdom encourages the use of theories, models and conceptual frameworks, yet their application is less evident in practice. This may be an artefact of reporting, indicating that prospective, primary research is needed to explore the real value of the KTA Framework and similar tools.

**Electronic supplementary material:**

The online version of this article (doi:10.1186/s13012-014-0172-2) contains supplementary material, which is available to authorized users.

## Background

Health professionals across the globe share the challenges of translating the best available evidence into actual health interventions in a timely way to provide the most effective care and service. The Knowledge to Action Framework [1] (‘the KTA Framework’) is a conceptual framework intended to help those concerned with knowledge translation deliver sustainable, evidence-based interventions. Knowledge translation has been defined as a process ‘*that includes synthesis, dissemination, exchange and ethically sound application of knowledge to improve…health…provide more effective health services and products and strengthen the health care system*’ [2].

Conceptual frameworks are recommended as a way of preparing for the multiple, dynamic and interactive factors that influence the uptake of evidence in practice [3]-[5]. Although the terms conceptual frameworks, theories and models are often used interchangeably, conceptual frameworks are broad and descriptive, whereas theories and models are more specific and amenable to hypothesis testing [6]. Conceptual frameworks provide a frame of reference for organising thinking, a guide for action and interpretation. Potential benefits from applying a conceptual framework include making the process of knowledge translation more systematic, with greater likelihood of changed practice and spread of evidence [4],[6]-[9]. Several conceptual frameworks are pertinent for implementation scientists, including Promoting Action on Research Implementation in Health Services (PARIHS) [5],[10] the Consolidated Framework for Implementation Research (CFIR) [3] as well as the KTA Framework [1].

### The Knowledge to Action Framework

The KTA Framework [1] was developed in Canada by Graham and colleagues in the 2000s in response to the confusing multiplicity of terms used to describe the process of moving knowledge into action [1]. They reviewed 31 planned action theories about the process of change. Most of the theories were interdisciplinary or from nursing and were published between 1983 and 2006 [11]. The findings informed their conceptual framework, which was intended to ‘*address the need for conceptual clarity in the KTA field and to offer a framework to help elucidate what we believe to be the key elements of the KTA process*’ [1] (p. 14)*.*

**Figure 1:**
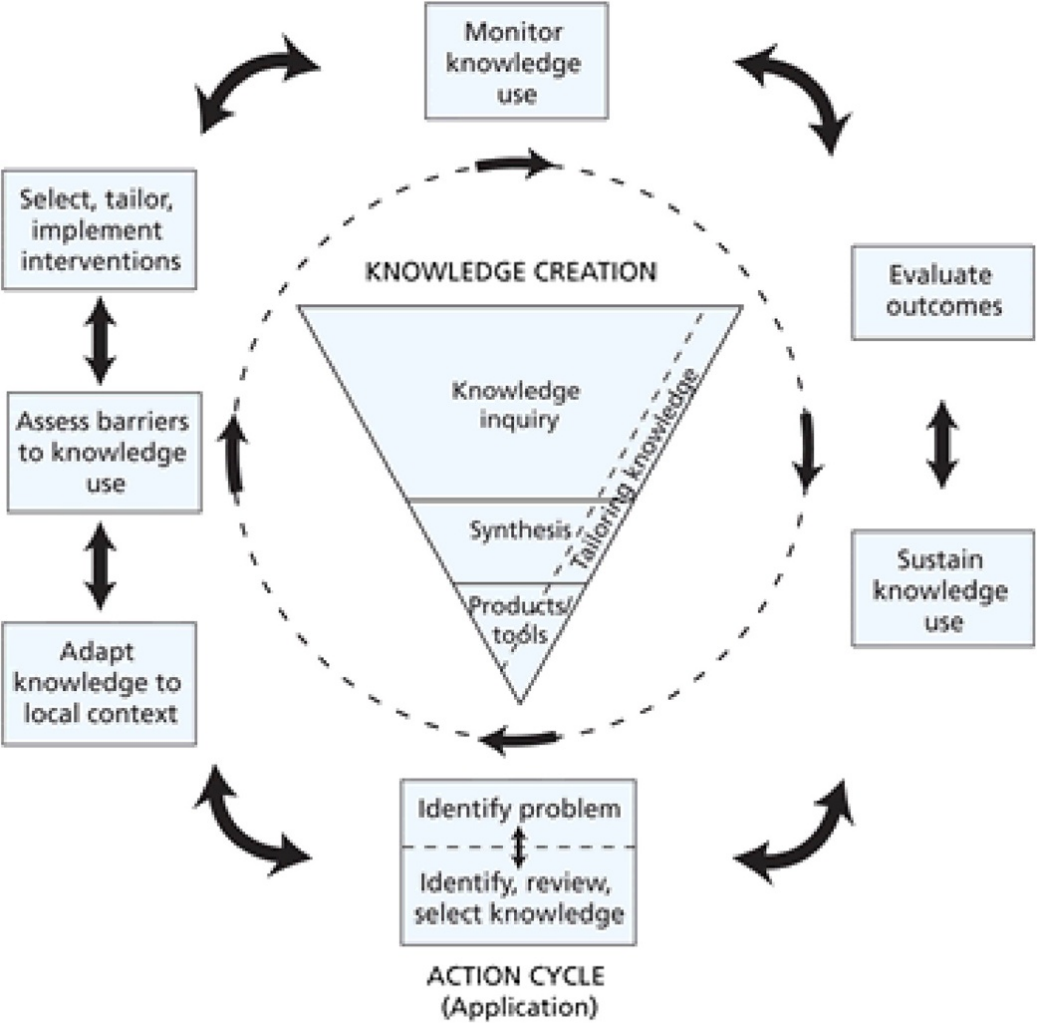
**The Knowledge to Action Framework.** From Graham I, Logan J, Harrison M, Strauss S, Tetroe J, Caswell W, Robinson N: Lost in knowledge translation: time for a map? *The Journal of Continuing Education in the Health Professions* 2006, 26, p. 19. Reprinted with permission from John Wiley and Sons.

The KTA Framework comprises two distinct but related components: (i) Knowledge Creation (represented by the funnel) surrounded by (ii) the Action Cycle (Figure 1). Each component involves several phases which overlap and can be iterative; Graham and colleagues [1] describe the phases as ‘*dynamic…can influence each other*’ (p. 20). Action phases may be carried out sequentially or simultaneously; knowledge phases may impact on the action phases. The Action Cycle outlines a process, representing the activities needed for knowledge to be applied in practice; knowledge is adapted to the local context, and barriers and facilitators to its use are explicitly assessed. Involvement of stakeholders, and tailoring knowledge to the needs of people who are going to use it, is crucial.

The National Institute for Health Research (NIHR) Collaboration for Leadership in Applied Health Research and Care for South Yorkshire (CLAHRC SY) used the KTA Framework to underpin a programme of knowledge translation work undertaken between 2008 and 2013 [12]. As part of this programme, the first author (BF) undertook a knowledge translation project that sought to identify examples of the use of the KTA Framework in practice. No existing systematic review was found on this subject. This review is designed to address this knowledge gap.

This review seeks to answer two questions: ‘Is the KTA Framework being used in practice?’ and ‘If so, how is the KTA Framework being used in practice?’ We were interested in the ‘real-life’ application of this conceptual framework to ‘real-world’ implementation challenges. A secondary concern was theory fidelity [13], which relates to how this conceptual framework was being used in practice, specifically whether the KTA Framework was articulated in a way that was true to the source paper [1].

## Methods

### Citation analysis and systematic review

We chose citation searching as our preferred method to identify reports of practical applications of a model or framework [14],[15]. Citation searching can circumvent the problems of variation in terminology (a retrieval problem) or uninformative abstracts (a reporting problem) typically encountered in topic-based searches of bibliographic databases. We sought to identify all reported citations of a particular reference irrespective of the context within which that reference had been used. Citation searching was further indicated in this review given that our scoping search, using the databases MEDLINE and CINAHL, had previously identified few abstracts reporting explicitly the use of the KTA Framework in practice.

The index citation for the source paper for the KTA Framework ‘Lost in knowledge translation: time for a map?’ by Graham and colleagues [1] was identified on each of three databases that offered functionality for citation searching. The databases were Scopus, Web of Science and Google Scholar. Citation searches were limited to the period from 2006 (the date of publication of the source paper) to July 2013. Duplicates between records from the databases were identified and the most complete record was retained for the subsequent sift process. Google Scholar references often included an incomplete text fragment rather than a more traditional abstract. However, supplementary hypertext links could be followed up from Google Scholar to either an abstract or, optimally, to the full text. For Web of Science and Scopus citations, full abstracts were identified. References to non-English language articles were removed. Google Scholar also enabled access to book chapters whereas Scopus and Web of Science only indexed peer-reviewed journal articles. All results were imported and combined into an Excel spreadsheet for review by two reviewers.

### Inclusion/exclusion criteria

All citations identified were screened by title and available information according to agreed criteria. Papers were excluded if they were not empirical real-life applications of the KTA Framework; if they were literature reviews; if they were only conceptual or descriptive papers (including discussion, commentaries or protocols); if they described a single knowledge translation strategy, such as a clinical practice guideline; or if they were not topically relevant. Given the incomplete abstracts obtained via Google Scholar, a ‘rule-out’ strategy was employed. Citations were excluded where there was insufficient information to make a judgment about inclusion/exclusion criteria. In practice, the implications of this decision were minimal as many Google Scholar records either duplicated full bibliographic records already identified from Web of Science or Scopus or contained at least sufficient detail in the title or in the limited text displayed (text fragment), to indicate whether the paper merited further consideration.

The inclusion criteria were based on an affirmative answer to two questions ‘Does the paper describe a KT project?’ and ‘Is the KTA Framework a fundamental guide to this project?’ The initial sift phase was carried out by one researcher (BF), with another researcher coding a proportion of these (II). Difficulties applying the exclusion criteria were discussed to ensure the inclusion/exclusion criteria were applied consistently. Any differences were discussed and resolved by consensus.

Full-text articles were obtained for any article coded for inclusion and for any articles that appeared relevant but where it was unclear whether the KTA Framework had been actually used in practice. Two researchers (BF and II) conducted an initial assessment of the full-text articles. This showed that the degree of usage varied from merely citing the KTA Framework to full integration into the study. We developed a taxonomy to categorise this variation (see Table 1). Included articles were re-examined and re-coded according to this more detailed classification. The taxonomy enabled us to refine the inclusion criteria to identify studies that reported explicit application of the KTA Framework. This produced a subset of studies that contained examples of using the KTA Framework in an integral way.

**Table 1:** **Taxonomy for application of the KTA Framework within included studies**

Category	Definition
Integrated	The KTA Framework was integral to the design, delivery and evaluation of the implementation activities.
Directed	The KTA Framework had influenced project design but with no examples given.
Adapted/combined	The KTA Framework had been modified or blended with another conceptual framework.
Informed	The KTA Framework had influenced the study in a general, non-specified way (e.g. in the Introduction or Discussion).
Referenced	The KTA Framework was cited with little or no further explanation.

### Data extraction

The data were extracted from the ‘integrated’ studies where the KTA Framework was applied in practice. Data extracted were mapped to each phase of Knowledge Creation and the Action Cycle (see Figure 1) by one researcher (BF) and then discussed with the whole team.

### Quality assessment

The quality of reporting was assessed using criteria adapted from Carroll and colleagues [16]. This involved checking whether the four elements were described. These were the question and study design, recruitment and selection and methods of data collection and analysis. We did not assess the research rigour of the individual knowledge translation projects. Rather, we focused on the application of, and theory fidelity to, the KTA Framework.

### Data analysis

Each article was scrutinised and mapped against the corresponding phases within the Knowledge Creation and Action Cycle components of the framework. Data for each phase were then synthesised across studies to help understand how the framework had been used in practice.

## Results

**Figure 2:**
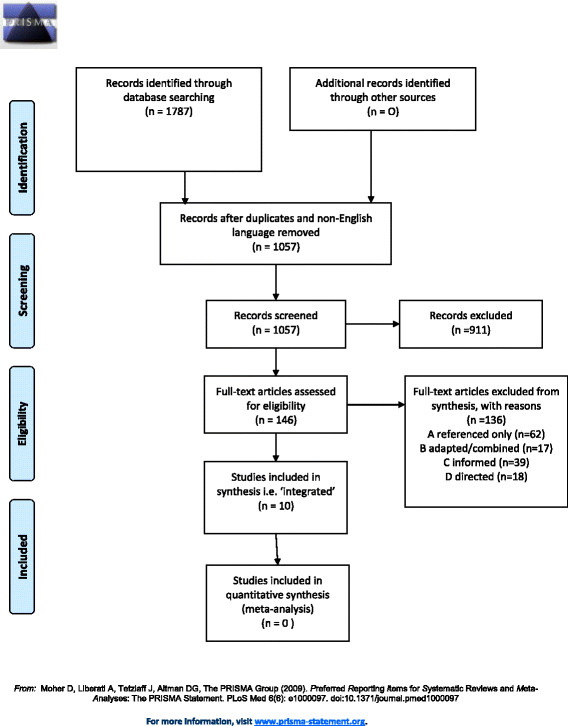
**PRISMA flow diagram of citation analysis and systematic literature review.**

The citation search for the original source paper [1] yielded 1,787 records. The result set was reduced to 1,057 records following removal of duplicate and non-English language records. Next, 911 records were excluded at the sift stage. Those excluded at this stage included literature reviews and conceptual, descriptive or commentary papers, papers describing a single knowledge translation strategy or not topically relevant. In other cases, we made an initial judgement, based on the abstract or a Google text fragment, that the paper was not about an empirical, real-life, knowledge translation or implementation project. A total of 146 papers were identified as attributing use of the KTA Framework. The search results are illustrated in Figure 2, the PRISMA flow chart.

### Attribution of use

The full text of the 146 papers was examined to establish the extent to which the use of the KTA Framework was attributed by the authors. The papers were coded according to the taxonomy in Table 1. Sixty two of the 146 papers (43%) were classified as ‘*referenced*’, meaning that the framework was cited with little, if any, further explanation (see Table 2). Thirty nine (27%) were categorised as ‘*informed*’ because the KTA Framework had influenced the project in a ‘non-specified’ way without citing examples of how it had been applied. Seventeen studies (12%) were noted as ‘*adapted* or *combined*’ where the KTA Framework had either been modified or blended with another conceptual framework. A further eighteen (12%) were categorised as ‘*directed*’ because the KTA Framework influenced the project design or helped to plan, structure or conceptualise what was done, but with no examples given. Only ten studies were integrated, signifying that the KTA Framework was integral to the design, delivery and evaluation of the implementation activities. Importantly, these integrated studies included examples to illustrate how the KTA Framework had been used as a guide. The following analysis focuses on results relating to these ten integrated studies.

**Table 2:** **Frequency and percentage of types of KTA Framework used**

Category	***n***(%)
Integrated	10 (7%)
Directed	18 (12%)
Adapted/combined	17 (12%)
Informed	39 (27%)
Referenced	62 (43%)
Total (*N*)	146

#### Characteristics of studies using the KTA Framework in an integrated way

Eight of the ten studies were conducted in Canada, one in the Democratic Republic of Congo [17] and one in Denmark [18]. Studies were published between 2007 and 2013. Nine were published in peer-reviewed journals with the exception being a Master of Science degree dissertation [19].

Each implementation study was very different (see Table 3). Two were concerned with public health or health promotion [17],[20]; three focused on clinical academic or nurse education [18],[21],[22]. Others related to specific conditions, such as stroke [23],[24], children with cerebral palsy and motor difficulties [25] and osteoarthritis [26]. The target audience included the public and patients, the families of older adults, health professionals, occupational therapists, speech and language therapists, physiotherapists and educationalists/academics within universities.

**Table 3:** **Characteristics of the studies reporting application of the KTA Framework in an integrated way**

	Authors, date of publication	Country of study	Focus of knowledge translation project	Target audience	Reported knowledge creation and/or action cycle
1	Bjrøk et al., 2013 [18]	Denmark	Nursing education; to get a model of skill performance taught as part of the nursing curriculum	Stakeholders within academic setting	Knowledge Creation and Action Cycle
2	Claude et al., 2012 [17]	Democratic Republic of Congo	Health promotion; prevention of spina bifida	Women in maternal and child health clinics	Action Cycle
3	Hua et al., 2012 [20]	Canada	Public health; Canadian Hypertension Education Programme	General public, health professionals and policymakers	Knowledge Creation and Action Cycle
4	Keyser, 2010^a^[19]	Canada	Community; to inform families about delirium in older adults	Families of older adults	Action Cycle
5	Molfenter et al., 2009 [23]	Canada	Evidence-based treatment for dysphagia patients post stroke	Speech and language therapists	Knowledge Creation and Action Cycle, mainly Action Cycle
6	Petzold et al., 2010 [24]	Canada	Unilateral spatial neglect post stroke	Occupational therapists	Knowledge Creation and Action Cycle
7	Russell et al., 2010 [25]	Canada	Rehabilitation setting; children with cerebral palsy; evidence-based paediatric measurement tools	Physiotherapists	Action Cycle
8	Stacey et al., 2009 [21]	Canada	Nursing education; integrating patient decision support within the undergraduate nursing curriculum	Academic stakeholders	Knowledge Creation and Action Cycle, mainly Action Cycle
9	Straus et al., 2008 [22]	Canada	Clinical/academicmentor scheme for clinician scientists	Stakeholders of the mentor scheme	Knowledge Creation and Action Cycle
10	Tugwell et al., 2007 [26]	Canada	Osteoarthritis	Consumers of health care including patients and clinicians	Knowledge Creation and Action Cycle

^a^Master of Science dissertation.

All ten studies reported using the Action Cycle. Seven described using both the Knowledge Creation and the Action Cycle components [18],[20]-[24],[27]. None described applying every phase of the KTA Framework.

#### Application of Knowledge Creation

Two studies reported using all phases of Knowledge Creation [20],[26] (see Table 4). Five studies applied one or more phases [18],[20],[22],[24],[26]. Tailoring knowledge was described in three studies [18],[20],[26]. Associated knowledge tools included summaries of evidence targeted at specific audiences, continuing health care education modules, information posted on websites and decision aids.

**Table 4:** **Application of the Knowledge Creation phases**

Authors, date of publication		Knowledge creation			
		Knowledge inquiry	Knowledge synthesis	Knowledge tools/products	Tailoring knowledge
1	Bjrøk et al., 2013^a^[18]	–	–	‘Instrumental’ manual; tailored through cooperation with stakeholders	✔
2	Claude et al., 2012 [17]	–	–	–	–
3	Hua et al., 2012 [20]	✔	✔	Summaries of evidence (one page to scientific analysis of changes to recommendations; slide decks; patients and provider tools; continuing health education modules; posted on website)	✔
4	Keyser, 2010^b^[19]	–	–	–	–
5	Molfenter et al., 2009^c^[23]	–	–	–	–
6	Petzold et al., 2010 [24]	✔	✔	–	–
7	Russell et al., 2010 [25]	–	–	–	–
8	Stacey et al., 2009^c^[21]	–	–	Development of exam questions, problem-based scenarios, materials in French; newsletter profiling different resources available	–
9	Straus et al., 2008 [22]	–	✔	–	–
10	Tugwell et al., 2007 [26]	✔	✔	One page summaries of evidence; ‘bottom lines’ highlighting risk/benefits; decision aids	✔

Key: – indicates that this phase was not named explicitly within the text.

^a^Reported ‘knowledge creation and tailoring’.

^b^Masters of Science dissertation, involving a literature review.

^c^These studies reported ‘knowledge creation’ but did not name individual phases within this component.

#### Application of the Action Cycle

All ten studies reported applying the Action Cycle, describing at least five of the seven possible phases (see Table 5). All undertook the first phase of ‘identifying the problem’. The least reported phase, or the phase carried out least often, was ‘sustain knowledge use’. Three studies illustrated each phase of the Action Cycle or explained their reasons for not doing so [18],[23],[25].

**Table 5:** **Application of the Action Cycle phases**

Authors, date of publication		Action Cycle component						
		Identify problem/identify review, select knowledge	Adapt knowledge to local context	Assess barriers to local use	Select, tailor, implement interventions	Monitor knowledge use	Evaluate outcomes	Sustain knowledge use
1	Bjrøk et al., 2013 [18]	✔	✔	✔	✔	✔	Reported plans	Reported plans
2	Claude et al., 2012 [17]	✔	–	✔	✔	✔	✔	Reported beyond scope
3	Hua et al., 2012 [20]	✔	✔	–	–	✔	✔	✔
4	Keyser, 2010 [19]	✔	✔	✔	✔	✔	✔	–
5	Molfenter et al., 2009 [23]	✔	✔	✔	✔	✔	✔	✔
6	Petzold et al., 2010 [24]	✔	–	✔	Reported plans	Reported plans	Reported plans	Reported plans
7	Russell et al., 2010 [25]	✔	✔	✔	✔	✔	✔	✔
8	Stacey et al., 2009 [21]	✔	✔	✔	✔	✔	✔	–
9	Straus et al., 2008 [22]	✔	✔	✔	✔	✔	✔	–
10	Tugwell et al., 2007 [26]	✔	–	✔	✔	✔	Reported plans	Reported plans

Key: – indicates that this phase was not named explicitly within the text.

Further information about how the different studies applied the phases within the Action Cycle are shown in Table 6. Each study demonstrates the particularity of implementation activities. Most studies illustrate how knowledge was adapted to the local context. Questionnaires, interviews, workshops, focus groups and needs assessment were used to identify barriers to change. Education was the most frequently employed strategy albeit in a variety of forms.

**Table 6:** **Detailed application of the phases with the Action Cycle**

	Authors, date of study	Problems identified and/or knowledge identified, reviewed, selected	How knowledge was adapted to local context	Methods of barrier assessment and barriers identified	Selection, tailoring and mplementation of interventions	Monitoring, evaluating and sustaining knowledge use
1	Bjrøk et al., 2013 [18]	Problem of qualifying nursing students in practical skill acknowledged by educators, researchers and students	‘Research in nursing skills group’ refined theoretical model; developed a supplement to the model; developed ‘heuristic’ devices	‘Research in nursing skills’ group created; identifies barriers; language of manual; lack of examples	Implementation skills centres and clinical practice; working with students, teachers, clinical supervisors	Monitoring as part of defined projects; process and outcome evaluation of the model in ongoing studies; sustaining knowledge use planned
2	Claude et al., 2012 [17]	Reviewed evidence that spina bifida is preventable through use of peri-conceptual maternal folate supplements; reviewed local surgical cases at one hospital	–	Knowledge, attitude and practices questionnaire (*n* =150 women attendees at prenatal consultations) and focus group identified limited use and awareness of folic acid, willingness to take it and to pay cost	Radio broadcasts and educational video	142 women completed questionnaires before and after video; 95% agreed video was understandable and should be widely disseminated. Short-term knowledge gain (pre/post-test) was found to have increased significantly. Evaluation and sustaining phases stated as beyond the scope of this project
3	Hua et al., 2012 [20]	‘Canadian Hypertension Education Program Recommendations Task Force’ identifies what is old but still important from evidence base and previous recommendations	Small group workshops; pilot testing of recommendations led by committee representative of the target health care professionals	‘Implementation task force’ ‘addresses barriers to knowledge use’; specific barriers not reported	Information and educational resources for health care practitioners, patients and policymakers (e.g. fact sheets, one-page summaries, post cards); ‘train the trainer’ programme to aid dissemination of materials (1-day course offered throughout the year in English and French, across Canada)	‘Outcomes Review Task Force’ examines national and provincial administrative data; for example, improved self-reported awareness of hypertension, use of multiple hypertensive agents, greater control of hypertension and improved age- and sex-standardised mortality rates
4	Keyser, 2010 [19]	Problem identified by focus group that families of elderly lacked understanding of delirium; literature reviewed and synthesised to ensure that information given was up to date	Existing educational resources gathered and appraised for level of appropriateness to lay population; best available chosen	Focus group with families identified limited awareness	Six education sessions and information package given to participants	‘Knowledge test’ before and after intervention; limitations of this approach to monitoring and evaluation and sustaining knowledge use discussed
5	Molfenter et al., 2009 [23]	‘Knowledge to action’ gap identified in swallowing rehabilitation; despite training, speech and language therapists not using recommended tool in practice; research team reviewed literature, designed treatment protocol	Adapted knowledge to the facility and individual clinicians—discussed potential application to their caseloads	Discussions between research team and clinicians identified time; appropriate patient selection; discomfort with the technology; competing priorities	Tailored support for clinicians; assistance to select appropriate patients on site; individualised training, ad hoc support and mentorship available via email, telephone and in person	Mentors monitored progress. Outcomes evaluated via qualitative interviews with clinicians; thematic analysis identified key themes. Sustaining knowledge use described as ongoing partnership between the research team and clinicians; clinicians continue to access support from mentors
6	Petzold et al., 2010 [24]	Two multicentre studies identified limited best practice for management of unilateral spatial neglect post stroke	Not reported	Focus groups with clinicians; interviews between researchers and clinicians; key themes re: barriers and facilitators identified; specific barriers not reported	Plans reported a for multimodal knowledge translation intervention, informed by barriers and facilitators identified	Plans reported for follow-up studies to address whether desired change has occurred
7	Russell et al., 2010 [25]	Survey revealed wide variation in practice of paediatric occupational therapists and physiotherapists treating re: best practice guidelines and measurement tools	‘Knowledge brokers’ given materials, for example, manual and instructional DVD for particular measurement tools, key articles, summaries and case scenarios; intranet site established; PowerPoint presentation for knowledge brokers to adapt as they wished	‘Support and barriers’ questionnaire completed by knowledge brokers re: organisational structure, resources, target therapists, the children and families and the measurement tools themselves; specific barriers not reported	Knowledge brokers in multiple sites—knowledge brokers recorded their activities, regular teleconferences and use of intranet to facilitate sharing of strategies	To monitor and evaluate outcomes, knowledge brokers and physiotherapists completed online survey re: knowledge and use pre and post intervention, and at 6, 12 and 18 months; semi-structured telephone interviews held with knowledge brokers, physiotherapists and centre administrators at 6 and 18 months
8	Stacey et al., 2009 [21]	Audit of nursing curriculum course identified lack of awareness and use of patient decision support resources (by nursing students and faculty members)	Plan for the integration of decision support throughout the nursing curriculum shared with key stakeholders; project advisory team established	Needs assessment with faculty members identified limited awareness , time pressures, limited resources in French and lack of instructional tools (e.g. case scenarios, exam questions assignments)	Faculty development activities to address identified barriers. For example, staff workshop, newsletter profiling different resources, exam questions, case scenarios, assignments created	Monitoring identified use of scenarios and assignments; lectures given; knowledge use observed amongst students in course assignments, seminars and exam questions; faculty members requested session on patient support plans for evaluation and sustaining knowledge use discussed
9	Straus et al., 2008 [22]	Need to provide adequate mentorship for clinical researchers identified by researchers and research funders	Workshop for funders, university administrators and clinician scientists to review evidence on mentorship tools	Workshop identified barriers as: lack of recognition of importance of mentoring at departmental/university levels; lack of educational intervention for mentors/mentees and difficulty finding mentors; lack of time and capacity	Written summary of key messages, local opinion leaders/academic detailing, website, newsletters targeting administrators, departmental chairs, researchers, mass media, chairs of research institutes	Monitoring identified development of mentorship facilitators and workshops. Plans reported to evaluate impact of adopted mentorship strategies, for example, repeating qualitative study re: experiences of mentorship; sustainability considered for example by trying to ensure ongoing participation of stakeholders, and qualitative evaluation—if the intervention not found to be effective, iterative cycle of design and testing to try and refine it
10	Tugwell et al., 2007 [26]	People with musculoskeletal conditions, family members and physicians were interviewed as part of another project—results indicated people wanted information about treatments and what they could do to help themselves, but health care providers had difficulty translating information into lay language	Evidence was adapted, for example, by describing situations when individuals may want to consider treatment, by presenting benefits and harms out of 100 people and decision aids	Interviews re: barriers identified the Internet as an important source of information but that physicians were still regarded as gatekeepers to information and services; literature re: barriers and facilitators also considered and identified lack of awareness, difficulty accessing them or limited time of physicians	Aimed to increase consumer awareness of and access to knowledge products/tools (patient decision aids on key websites and given to key helplines/call centres); on website for physicians to access and print out decision aids	Describe plans to monitor use of these tools; basic evaluation of website visits and a web survey completed. Authors discuss challenges of evaluating outcome and sustaining knowledge use and other work related to this

Key: – indicates that this phase was not named explicitly within the text.

#### Quality assessment

Quality assessment of the integrated studies proved problematic given that many papers did not report research questions, traditional research designs or recruitment and selection of participants as required by the criteria [16]. Rather, papers typically stated aims and objectives, which often related to closing evidence/knowledge-practice gaps. Papers could be scrutinised according to how their aims and objectives had been operationalised. On this basis, all ten studies were judged as having described their projects clearly. Four of the ten studies [17],[19],[21],[23] reported gaining ethical approval, suggesting that these had been independently characterised as research.

## Discussion

The KTA Framework [1] is being used in practice with varying degrees of completeness and theory fidelity when the conceptual framework is integrated into the implementation project. It is one of the most frequently cited conceptual frameworks for knowledge translation. A citation search of three databases tracking the source paper ‘Lost in knowledge translation: time for a map?’ identified 1,787 records between 2006 and July 2013. Some indication of the impact of this article can be gained from constructing a ‘normalised citation count’ for comparison purposes. The source paper was cited four times more frequently than the next highest cited article from the same journal published in the same year. It was cited 470 unique times across all databases. However, citation figures do not reflect how this conceptual framework has actually been applied in practice. We initially included 146 studies that reported using the KTA Framework. Closer examination revealed that usage varied considerably, ranging from simple attribution via a reference through to being integral to most aspects of the implementation work. Only ten studies reported and gave supportive examples of incorporating the KTA Framework in an integrated way. All these described the Action Cycle and seven referred to Knowledge Creation, articulating the KTA Framework in a way that was true to the source paper [1].

There was substantial variation in the setting and target audience for each documented change, the methods used to apply the KTA Framework and the terminology employed to report the phases within Knowledge Creation and the Action Cycle. This reflects the spread of the framework across a range of settings, to different health care services and systems and larger scale and smaller projects. The KTA Framework was adapted to different health service settings and resources, indicating a good fit for the diversity of real-world health care. However, the target audiences were primarily patients, the public and the nursing and allied health professions.

Most studies (8/10) were conducted in Canada where the KTA Framework originated. This frequency of use in Canada could be explained by the influence of national Canadian networks and dissemination activities. Perhaps more significantly, the KTA Framework is associated with the Canadian Institutes of Health Research [27]. Estabrooks and colleagues [28] comment that the Canadian research funding organisation had adopted the KTA Framework to guide knowledge translation, deploying specific grant mechanisms ‘*to ensure involvement of knowledge users with researchers throughout the research process*’ (p. 2). This may explain the varying degrees to which the framework was used.

The KTA Framework was enacted in a variety of ways, from informing to full integration, showing flexibility of use and that it can fit local circumstances and need. Use at a ‘lighter’ level through adapting or combining with other conceptual frameworks could be considered a strength, in that the KTA Framework offers the flexibility to be combined with other frameworks, being responsive to facilitating practitioner preferences and context-specific needs. It is important to note that Graham and colleagues have continued to publish on applications of the framework and the multiple factors to be considered [29]. In addition, this suggests conceptual frameworks do not have to be mutually exclusive.

Each study applied the framework in an idiosyncratic way. None reported using every phase of the KTA Framework. Only four studies detailed Knowledge Creation, yet existing knowledge was utilised in the other studies to identify knowledge-practice gaps, or as part of the Action Cycle. This flexibility was intended, as Graham and colleagues [1] state the framework can ‘…*also accommodate different phases being accomplished by different stakeholders and groups (working independently of each other) at different points in time*’ (p. 18). Implementation researchers and health professionals can learn from this flexibility. It may be useful for them to consider the extent to which they wish to follow or be guided by a conceptual framework before embarking on a knowledge translation project, especially regarding outcome measures because ‘*....the focus of knowledge into action is ultimately to enhance health status*’ [1 p. 18]. The Action Cycle was reported in all the integrated examples, illustrating theory fidelity in this specific subset of studies. The prevalence of the Action Cycle may simply reflect the cyclical nature of the change process evident in other common, quality improvement tools such as the ‘Plan, Do, Study, Act’ cycle [30]. Also, this conceptual framework may appeal because of a lack of jargon and a simple diagram depicts the knowledge translation process.

The integrated studies described different ways of integrating the KTA Framework, particularly the Action Cycle. Most studies focused on improving knowledge or awareness, supporting what we know about the preponderance of professional or educational knowledge translation strategies within interventions aiming to promote the uptake of evidence [31]. Nine of the studies reported assessing barriers to change [17]-[19],[21]-[26]. Knowledge-related barriers [32] were the most common, indicating the appropriateness of using educational strategies to address such barriers. Yet the albeit limited, evidence available indicates that bringing information close to the point of decision-making (such as using reminders or decision support tools) is likely to be more effective than using more traditional educational strategies (such as study, teaching or training) to try to address barriers and change practice [31],[33]. Only one study [26] reported using decision support tools as a knowledge translation strategy, although it is possible others did not report all the details of strategies they used to promote the adoption of their interventions. Knowledge translation strategies can include elements such as linkage and exchange, audit and feedback, informatics and patient-mediated and organisational interventions [29]. However, none of these knowledge translation strategies, designed to target different barriers, featured in the included studies, with one exception. Russell and colleagues [25] describe use of a knowledge broker, an example of a ‘linkage and exchange’-type strategy.

The importance of organisational or external factors and the ability to influence them is well recognised [1],[3],[33]-[35]. Authors identified many barriers relating to ‘environmental’ factors [32] such as lack of time and/or resources. Generally, it was difficult to ascertain whether the methods used captured, and indeed subsequently addressed, the full range of barriers. It may be that when people are consulted, they identify those barriers that they feel able to influence, such as knowledge or awareness, rather than organisational barriers, which could be perceived as more problematic or more distant. An exception was the study by Russell and colleagues [25] who reported using a questionnaire to assess a range of potential barriers and facilitators. Molfenter and colleagues [23] describe a strategy to assist clinicians with patient selection which could be interpreted as seeking to influence organisational barriers such as ‘competing priorities’, as well as addressing knowledge-related barriers. Légaré [32] recommends using established taxonomies developed for barriers and facilitators within knowledge translation projects. Our findings support this proposal. We further identified a need to use taxonomies when analysing or evaluating knowledge translation strategies [33],[34]. This study also reinforces the importance of reporting standards [8],[36], such as the new TIDieR checklist [37] to facilitate more explicit reporting of implementation studies and their subsequent inclusion in systematic reviews.

The monitoring, outcomes or sustaining phases of the Action Cycle were less often described, although three noted their plans for doing so [18],[24],[26]. This may reflect a publication bias, between reporting process and outcomes. Claude and colleagues [17] stated that these phases were beyond the scope of their project. Such work may require additional funding for longer term monitoring or strategies to sustain knowledge use over time. It may also be a reflection of the challenges for defining and reporting outcomes for knowledge translation projects. Tugwell and colleagues [26] highlight this particular challenge, commenting that most outcomes in arthritis research are about pain and function. In contrast, they wanted to evaluate the impact of their intervention on people’s ability to understand their choices and make informed decisions about treatment. Consequently, we recommend that the phase ‘select, tailor, implement intervention’ be amended to include ‘define and select outcomes and knowledge use measures’, as a prompt to those using the KTA Framework to specify such outcomes when selecting which knowledge translation strategies to use.

A notable feature of the KTA Framework is the development of knowledge tools or products as part of Knowledge Creation. Most studies created a range of products, either as part of Knowledge Creation [18],[20],[21],[26] or the Action Cycle [21],[23],[24]. Interestingly, a recent evaluation of the Canadian Institutes of Health Research (CIHR) Knowledge Translation Funding Program [38] presented results relating to knowledge translation products, academic outputs and capacity building together, giving the appearance, in our interpretation, that these different dimensions may be regarded as equally important.

Interest in using systematic literature reviews to investigate theories, models and conceptual frameworks has increased in recent years [7],[39]. Yet, this method may prove challenging, often because of limited and imprecise reporting. Davies and colleagues [40] note that less than 6% of 235 studies, albeit published before 1998, explicitly used theories of behaviour or behaviour change. They and we resorted to a simple taxonomy to describe the level and type of usage based on explicit reporting. A coding scheme, with 19 categories for theory use for behaviour change interventions, ranging from mentioned but not demonstrated, right through to theory refinement, has been developed [41]. This scheme may be useful for similar studies examining theory use. However, our review focused primarily on examining how a conceptual framework had been used in practice. Using the framework itself as a device through which to examine how it had been used seemed an appropriate and pragmatic approach for our purposes.

Bartholomew and Mullen [42] suggest that the ‘*prevailing wisdom in the field of health-related behaviour change is that well-designed and effective interventions are guided by theory*’ (p. S20). Others argue that the effectiveness and generalisability of implementation studies are hindered by weak theoretical underpinnings [40],[43],[44]. Our review, and similar studies [39],[40],[43]-[45], consistently comments on the limited, haphazard use of theory, even though theories can be applied in many different ways [41]. Primary studies, exploring the direct experience and perceptions of different stakeholders in implementation projects, which have been guided by conceptual frameworks, or theories, would add to our understanding of the utility and impact of these tools. A few authors have attempted this, such as McEvoy and colleagues [43] who examined benefits reported by authors using the Normalization Process Theory. A prospective design would strengthen research studies.

Future research could examine the studies which we categorised as using the KTA Framework to a lesser degree, perhaps for conceptual or persuasive reasons rather than instrumentally. It would be interesting to review the conceptual papers we excluded to explore how they were influenced by the framework and informed the development of conceptual frameworks more generally. Exploring the impact of the KTA Framework, and other conceptual frameworks, on patients and the public in terms of health improvement and outcomes would also be worthwhile, as would exploring their involvement in the application of the framework, not just as recipients of services but as key stakeholders in each phase.

### Limitations

Our study had several limitations. Firstly, there is a risk of bias and subsequent error given the lead author conducted most of the initial screening, all data extraction and synthesis. This was necessary given the limited resources available to support the review process. It is possible some potentially relevant studies were excluded during the initial sift stage. Several strategies were used to counter this risk. For example, difficulties in applying exclusion/inclusion criteria were discussed by the team and all subsequent decisions were then resolved by consensus. The final list of integrated studies was agreed by two authors (BF and II), and the synthesis was discussed in detail by the team. Initial ‘screening out’ by title and abstract on the basis of partial information from Google Scholar may also have excluded relevant studies. This is a limitation of using Google Scholar for citation searches. We acknowledge that there are multiple choices available when conducting citation searches. In the absence of formal comparisons of citation search techniques, we decided to operationalise citation searching using Google Scholar. Although there may be some small variation in the actual sets of references retrieved by different citation searches, we have no reason to believe that we have systematically under- or over-represented particular types of studies in our sample. Non-English language studies were excluded, reducing access to examples of applying the KTA Framework published in other languages. This is noteworthy given Canadian research may be published in French language journals. Further, the time frame of the citation searches only captures work up to a certain point (from the date of publication of the source paper to July 2013). We recognise that assimilation and utilisation of an influential framework is a continuous process and that we have employed essentially a cross-sectional method to survey the literature. However, the method is replicable and could be repeated to update the review in future.

Selection bias is another limitation given that we aimed to identify papers reporting usage of the KTA Framework rather than considering or comparing with other conceptual frameworks. This reflected our focus on whether the KTA Framework is used in practice and, if so, how it is applied.

The search strategy was limited to citation searching of three databases. We did not follow up references (including book chapters) or contact authors of included or excluded studies. Neither did we seek out grey literature or search specific thesis/dissertation databases. Citation searching seeks to optimise sensitivity and specificity when seeking to identify reports of practical applications of a model or framework. Our experience certainly confirms that this search method circumvents the problems of variation in terminology typically encountered in topic-based searches of bibliographic databases. However, as shown by our study, citation searching in isolation from citation analysis—the detailed examination of full-text—is unable to discriminate between where a model is simply mentioned ‘in passing’ or even for ‘cosmetic’ reasons and where it represents an explicit intellectual and conceptual contribution.

We acknowledge that faced with the same task, another review team might choose to include papers reporting a single knowledge translation strategy. We did not include the many papers about clinical practice guidelines for two interrelated reasons. Firstly, we were interested in the practical application of the KTA Framework and thus in identifying papers where the KTA Framework appeared to be a fundamental guide to the work reported. Secondly, multifaceted strategies are more likely to be successful than a single strategy [27],[46],[47], as they target different barriers [40], which reflects our interest in the real-world use of the KTA Framework, where it is probably impossible to control and isolate one strategy [48]. However, it is worth noting that clinical guidelines could be categorised as knowledge tools/products and/or implementation strategies depending on if, and how, the guideline features in the framework.

Similarly, we acknowledge that some readers may be interested in precise reasons for exclusions. We have chosen to report the aggregate number of excluded papers on the PRISMA flow diagram (Figure 2). Due to overlapping categories (e.g. review papers, conceptual or descriptive papers and those describing a single knowledge translation strategy or not topically relevant), we excluded against a single criterion, even when multiple criteria applied, as practical considerations rendered it unnecessary to exhaustively document all possible reasons for exclusion for each paper.

Inevitably, decisions about including or excluding studies were reliant on subjective judgements about whether the KTA Framework had been reported in an integrated way, or not. A continuum of usage, ranging from referenced to integrated, was developed to aid this process. Judging between informed and directed was difficult, suggesting further refinement of the categories is required.

Data extraction and presenting results according to the phases were also challenging, given that the framework is dynamic, and can be non-sequential with overlap between phases [1]. There were occasions when we sensed that aspects of Knowledge Creation and Action Cycle had been done or combined, but as this was not explicit, we excluded these data. For example, the creation of websites, interactive e-learning modules, training packages and a protocol were reported as part of the Action Cycle [19],[23],[24], yet they could be knowledge tools/products. This reinforces the importance of theory fidelity and that authors refer to established taxonomies or reporting standards [36],[37],[40],[41] so we can understand how conceptual frameworks, theories and models are really used to guide practice or inform research.

## Conclusion

This citation analysis and systematic review investigated the practical application of a well-established conceptual framework—the KTA Framework [1]. We found that the framework is being used in practice, to varying degrees of completeness, and with theory fidelity when reported as integral to the implementation effort.

Citation searching for the original reference by Graham and colleagues [1] optimised the sensitivity and specificity of the search strategy. It was cited 470 unique times across the three databases, indicating the bibliometric impact of the source paper. However, we were interested in the real-world application of the KTA Framework as a guide to implementation activities in health care and whether it was used in a way that was true to the framework. The authors’ attribution of use was variable, with most merely referencing the framework or describing how it was used to inform or structure projects. Only 10 of 146 papers reported direct use, with examples demonstrating that the framework was integral to their study. In these studies, the KTA Framework appeared to provide a practical yet flexible guide to getting research findings into practice, allowing it to be applied in idiosyncratic ways. This illustrates the adaptability of the conceptual framework to different health care settings and topics. The KTA could also be used as a literacy device to structure reporting of the framework.

Conventional views support the use of theories, models and conceptual frameworks to underpin the process of change, yet in practice, their application seems more limited [40],[43]-[45]. This may be an artefact of reporting: few studies justify or explain their theoretical stance; even fewer do so using the appropriate constructs or with theory fidelity. This presents an interpretation challenge both for those seeking to learn from such projects and systematic reviewers. Understanding if and how these theoretical tools add value to implementation endeavours and outcomes merits further research.

## Authors’ information

BF is a researcher at the School of Health and Related Research (ScHARR), University of Sheffield, and also an occupational therapist. AB is Reader in Evidence Based Information Practice at the School of Health and Related Research (ScHARR), University of Sheffield, where he leads on systematic review and evidence synthesis activities. II is an occupational therapist. Between 2009 and 2013, she was a Knowledge Translation Project Lead with the NIHR CLAHRC SY. II provided BF with clinical supervision for her MSc dissertation. KG is a professor of nursing research at the University of Sheffield and Sheffield Teaching Hospitals NHS Foundation Trust where she leads the Translating Knowledge into Action theme of NIHR CLAHRC for Yorkshire and Humber. From 2008 to 2013, she led the Translating Knowledge into Action theme of NIHR CLAHRC SY.

## Authors’ original submitted files for images

Below are the links to the authors’ original submitted files for images. Authors’ original file for figure 1 Authors’ original file for figure 2

## Abbreviations

CIHR: Canadian Institutes of Health Research
CLAHRC SY: Collaboration for Leadership in Applied Health Research and Care for South Yorkshire
CLAHRC YH: Collaboration for Leadership in Applied Health Research and Care for Yorkshire and Humber
KTA Framework: Knowledge to Action Framework
NIHR: National Institute for Health Research

## References

[1] Graham I, Logan J, Harrison M, Straus S, Tetroe J, Caswell W, Robinson N (2006). Lost in knowledge translation: time for a map?. J Contin Educ Health Prof.

[2] More About Knowledge Translation at CIHR—CIHR. -[http://www.cihr-irsc.gc.ca/e/39033.html],

[3] Damschroder LJ, Aron DC, Keith , Rosalind E, Kirsh SR, Alexander JA, Lowery JC: Fostering implementation of health services research findings into practice: a consolidated framework for advancing implementation science. Implement Sci. 2009, 4:10.1186/1748-5908-4-50 PMC2736161 19664226

[4] Eccles M, Grimshaw J, Walker A, Johnston M, Pitts N (2005). Changing the behavior of healthcare professionals: the use of theory in promoting the uptake of research findings. J Clin Epidemiol.

[5] Rycroft-Malone J (2004). The PARIHS framework: a framework for guiding the implementation of evidence based practice. J Nurs Care Qual.

[6] Rycroft-Malone J, Bucknall T (2010). Models and Frameworks for Implementing Evidence-Based Practice.

[7] Ilott I, Gerrish K, Booth A, Field B (2013). Testing the consolidated framework for implementation research on health care innovations from South Yorkshire. J Eval Clin Pract.

[8] Helfrich CD, Damschroder LJ, Hagedorn HJ, Daggett GS, Sahay A, Ritchie M, Damush T, Guihan M, Ullrich PM, Stetler CB (2010). A critical synthesis of literature on the promoting action on research implementation in health services (PARIHS) framework. Implement Sci.

[9] Tabak RG, Khoong EC, Chambers DA, Brownson RC (2012). Bridging research and practice: models for dissemination and implementation research. Am J Prev Med.

[10] Rycroft-Malone J, Harvey G, Kitson A, McCormack B, Seers K, Titchen A (2002). Getting evidence into practice: ingredients for change. Nurs Stand.

[11] Graham ID, Tetroe J (2007). Some theoretical underpinnings of knowledge translation. Acad Emerg Med.

[12] Ilott I, Gerrish K, Bray K, Laker S: Tackling the implementation challenge: bridging thegapbetweenresearchandpractice. [http://www.rcn.org.uk/__data/assets/pdf_file/0014/512600/2013_RCN_research_W06.pdf]

[13] Crosby R, Noar SM (2010). Theory development in health promotion: are we there yet?. J Behav Med.

[14] Finfgeld-Connett D, Johnson ED (2013). Literature search strategies for conducting knowledge-building and theory-generating qualitative systematic reviews. J Adv Nurs.

[15] Booth A, Harris J, Croot E, Springett J, Campbell F, Wilkins E (2013). Towards a methodology for cluster searching to provide conceptual and contextual “richness” for systematic reviews of complex interventions: case study (CLUSTER). BMC Med Res Methodol.

[16] Carroll C, Booth A, Cooper K (2011). A worked example of “best fit” framework synthesis: a systematic review of views concerning the taking of some potential chemopreventive agents. BMC Med Res Methodol.

[17] Claude KM, Juvenal KL, Hawkes M (2012). Applying a knowledge-to-action framework for primary prevention of spina bifida in tropical Africa. Matern Child Nutr.

[18] Bjørk IT, Lomborg K, Nielsen CM, Brynildsen G, Frederiksen A-MS, Larsen K, Reierson IÅ, Sommer I, Stenholt B (2013). From theoretical model to practical use: an example of knowledge translation. J Adv Nurs.

[19] Keyser SE (2010). The Implementation of a Delirium Knowledge Translation Project for Families of the Elderly.

[20] Hua D, Carter S, Bellerive J, Allu SO, Reid D, Tremblay G, Lindsay P, Tobe SW (2012). Bridging the gap: innovative knowledge translation and the Canadian hypertension education program. Can J Cardiol.

[21] Stacey D, Higuchi K, Menard P (2009). Integrating patient decision support in an undergraduate nursing curriculum: an implementation project. Int J Nurs Educ Scholarsh.

[22] Straus S, Graham I (2008). Development of a mentorship strategy: a knowledge translation case study. J Contin Educ Health Prof.

[23] Molfenter S, Ammoury A, Yeates E, Steele C (2009). Decreasing the knowledge to action gap through research-clinical partnerships in speech-language pathology. Can J Speech-Language Pathol Audiol.

[24] Petzold A, Korner-Bitensky N, Menon A (2010). Using the knowledge to action process model to incite clinical change. J Contin Educ Health Prof.

[25] Russell DJ, Rivard LM, Walter SD, Rosenbaum PL, Roxborough L, Cameron D, Darrah J, Bartlett DJ, Hanna SE, Avery LM (2010). Using knowledge brokers to facilitate the uptake of pediatric measurement tools into clinical practice: a before-after intervention study. Implement Sci.

[26] Tugwell PS, Santesso NA, O’Connor AM, Wilson AJ (2007). Knowledge translation for effective consumers. Phys Ther.

[27] Kennedy CC, Ioannidis G, Giangregorio LM, Adachi JD, Thabane L, Morin SN, Crilly RG, Marr S, Josse RG, Lohfeld L, Pickard LE, King S, van der Horst M-L, Campbell G, Stroud J, Dolovich L, Sawka AM, Jain R, Nash L, Papaioannou A (2012). An interdisciplinary knowledge translation intervention in long-term care: study protocol for the vitamin D and osteoporosis study (ViDOS) pilot cluster randomized controlled trial. Implement Sci.

[28] Estabrooks CA, Teare GF, Norton PG (2012). Should we feed back research results in the midst of a study?. Implement Sci.

[29] Straus S, Tetroe J, Graham ID (2013). Knowledge Translation in Health Care. Moving from Evidence to Practice.

[30] Taylor MJ, McNicholas C, Nicolay C, Darzi A, Bell D, Reed JE (2013). Systematic review of the application of the plan-do-study-act method to improve quality in healthcare. BMJ Qual Saf.

[31] Wensing M, Bosch M, Grol R, Straus S, Tetroe J, Graham ID (2009). Selecting, tailoring, and implementing knowledge translation interventions. Knowledge Translation in Health Care: Moving from Evidence to Practice.

[32] Légaré F, Straus S, Tetroe J, Graham I (2009). Assessing barriers and facilitators to knowledge use. Knowledge Translation in Health Care: Moving from Evidence to Practice.

[33] EPOC Taxonomy of Professional and OrganisationalInterventions.[https://epoc.cochrane.org/sites/epoc.cochrane.org/files/uploads/EPOC%20Taxonomy%20of%20Interventions%202002.pdf]

[34] Kitson AL, Rycroft-Malone J, Harvey G, McCormack B, Seers K, Titchen A (2008). Evaluating the successful implementation of evidence into practice using the PARiHS framework: theoretical and practical challenges. Implement Sci.

[35] McCormack B, Kitson A, Harvey G, Rycroft-Malone J, Titchen A, Seers K (2002). Getting evidence into practice: the meaning of ‘context’. J Adv Nurs.

[36] Davidoff F, Batalden P, Stevens D, Ogrinc G, Mooney SE (2009). Publication guidelines for quality improvement studies in health care: evolution of the SQUIRE project. BMJ.

[37] Hoffmann TC, Glasziou PP, Boutron I, Milne R, Perera R, Moher D, Altman DG, Barbour V, Macdonald H, Johnston M, Lamb SE, Dixon-Woods M, McCulloch P, Wyatt JC, Chan A-W, Michie S (2014). Better reporting of interventions: template for intervention description and replication (TIDieR) checklist and guide. BMJ.

[38] McLean R, Tucker J: Evaluation of CIHR’s Knowledge Translation Funding Program—CIHR.[http://www.cihr-irsc.gc.ca/e/47332.html#a2.1].

[39] Grimshaw JM, Eccles MP, Lavis JN, Hill SJ, Squires JE (2012). Knowledge translation of research findings. Implement Sci.

[40] Davies P, Walker AE, Grimshaw JM (2010). A systematic review of the use of theory in the design of guideline dissemination and implementation strategies and interpretation of the results of rigorous evaluations. Implement Sci.

[41] Michie S, Prestwich A (2010). Are interventions theory-based? Development of a theory coding scheme. Health Psychol.

[42] Bartholomew LK, Mullen PD (2011). Five roles for using theory and evidence in the design and testing of behaviour change interventions. J Public Health Dent.

[43] McEvoy R, Ballini L, Maltoni S, O Donnell CA, Mair FS, Macfarlane A (2014). A qualitative systematic review of studies using the normalization process theory to research implementation processes. Implement Sci.

[44] Thomas A, Menon A, Boruff J, Rodriguez AM, Ahmed S (2014). Applications of social constructivist learning theories in knowledge translation for healthcare professionals: a scoping review. Implent Sci.

[45] Ullrich PM, Sahay A, Stetler CB (2014). Use of implementation theory: a focus on PARIHS. Worldviews Evid Based Nurs.

[46] Menon A, Korner-Bitensky N, Kastner M, McKibbon KA, Straus S (2009). Strategies for rehabilitation professionals to move evidence—based knowledge into practice: a systematic review. J Rehabil Med.

[47] Powell BJ, McMillen C, Proctor E, Carpenter CR, Griffley RT, Bunger AC, Glass JE, York JL (2012). A compilation of strategies for implementing clinical innovations in health and mental health. Med Care Res Rev.

[48] Rycroft-Malone J (2012). Implementing evidence-based practice in the reality of clinical practice. Worldviews Evid Based Nurs.

