# Supplementary figures and images for: Using the Knowledge to Action Framework in practice: a citation analysis and systematic review
Source: Implement Sci. 2014 Nov 23;9:172. doi: 10.1186/s13012-014-0172-2 (PMC4258036; doi:10.1186/s13012-014-0172-2)

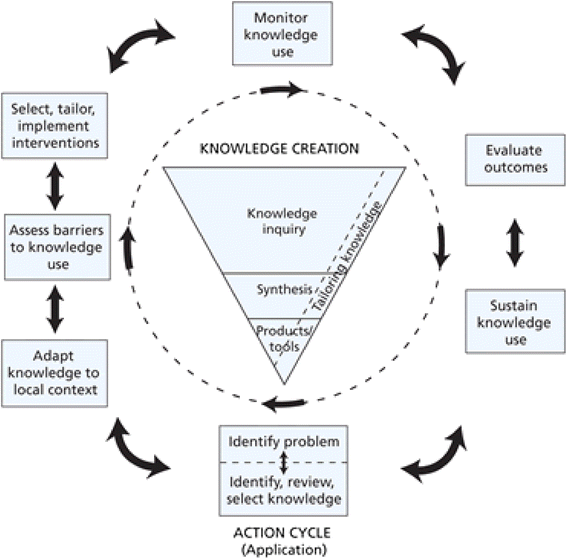

Supplement: Supplementary file 1 — Authors’ original file for figure 1 [file 13012_2014_172_MOESM1_ESM.gif]

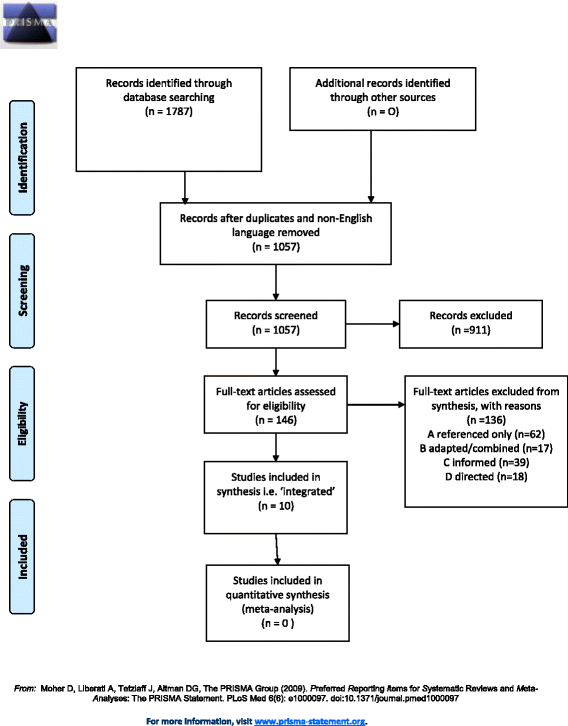

Supplement: Supplementary file 2 — Authors’ original file for figure 2 [file 13012_2014_172_MOESM2_ESM.gif]
